# Advancements in Vaccinology Against Infectious Hematopoietic Necrosis Virus (IHNV): From Traditional Methods to Next-Generation Strategies

**DOI:** 10.3390/vaccines14040314

**Published:** 2026-03-31

**Authors:** Wen Shi, Diqiu Liu

**Affiliations:** 1 College of Animal Science and Technology, Northeast Agricultural University, Harbin 150030, China; wenshi@neau.edu.cn; 2 State Key Laboratory of Animal Disease Control and Prevention, Harbin Veterinary Research Institute, Chinese Academy of Agricultural Sciences, Harbin 150069, China

**Keywords:** systematic review, IHNV, vaccinology, next-generation, Genetically Modified Organisms (GMOs), mucosal immunity, long-term control

## Abstract

Background: Infectious hematopoietic necrosis virus (IHNV), a rhabdovirus classified within the genus *Novirhabdovirus*, continues to be one of the most detrimental pathogens impacting salmonid aquaculture on a global scale. Notable for inducing high mortality rates among fry and fingerlings, IHNV represents a substantial threat to the economic stability of the aquaculture industry. This review offers an in-depth analysis of the contemporary advancements in IHNV vaccine development. Methods: We assess the efficacy and immunological mechanisms of traditional vaccine platforms, including inactivated and live-attenuated vaccines, while emphasizing the groundbreaking success of DNA vaccines, particularly those encoding the viral glycoprotein (G). Although nucleic acid-based therapies provide high levels of protection, they face logistical challenges related to delivery and regulatory obstacles associated with Genetically Modified Organisms (GMOs). Additionally, we examine emerging “next-generation” platforms, such as viral vector vaccines, subunit proteins produced in yeast or plant systems, and RNA-based technologies. We critically analyze technical bottlenecks, including the lack of efficient mucosal delivery systems and the limited understanding of long-term cellular memory in teleosts. Results: We propose future research directions that emphasize the development of multivalent formulations and the incorporation of molecular adjuvants to augment mucosal immunity. Conclusions: This synthesis seeks to integrate fundamental viral pathogenesis with applied immunology to develop a strategic framework for the sustainable, long-term management of IHNV in global salmonid populations.

## 1. Introduction

Infectious hematopoietic necrosis virus (IHNV) represents one of the most significant biological threats to the global sustainability of salmonid aquaculture [1]. It serves as the primary limiting factor for both freshwater and marine production systems [2]. IHNV is a member of *Novirhabdovirus salmonid*, the genus *Novirhabdovirus*, the subfamily *Gammarhabdovirinae*, and the family *Rhabdoviridae*, and it has a single-stranded negative-sense RNA genome of around 11,000 nt [1,2]. The virus mainly infects the hematopoietic tissues of susceptible hosts, the anterior kidney, and the spleen, causing systemic necrosis, exophthalmia, and abdominal distension [2]. The virus was first identified in the 1950s on the North Pacific Coast of North America [3,4]. Its ecological niche has since expanded because of the movement of eggs and fish by humans, as observed in Europe and East Asia [2]. The management of IHNV is complicated by the presence of asymptomatic carriers, which facilitate horizontal transmission via waterborne sheds and vertical transmission via contaminated ovarian fluids [5]. Consequently, the virus is essentially always present in the environment [6].

The economic ramifications of IHNV outbreaks in aquaculture for which these pathogens are responsible range from direct losses of fish, increased trade restrictions, heightened veterinary oversight, and costs associated with emergency culling and disinfection [2,7]. In the high-density cultivation practices of the Pacific Northwest and rising aquaculture production in China and Chile, IHNV is considered a notifiable disease of the World Organization for Animal Health (WOAH) due to its significant impact on international trade [8,9]. The pathogenesis of IHNV has been attributed to its molecular structure, which includes a non-virion (NV) gene that is exclusive to this genus [10,11]. It is believed that the NV gene plays a role in suppressing innate or non-specific immunity. IHNV induces a state of immunosuppression by inhibiting the induction of Type I Interferon (IFN I) and downstream Myxovirus resistance (Mx) proteins, thereby enabling rapid viral multiplication [12,13]. The outcome of the infection is determined by an arms race between the viral NV protein versus the host teleost immune system reaction. Moreover, the high mutation rate associated with RNA viruses enables IHNV to develop adaptations to new pressures from the environment and host [14]. This evolutionary process can lead to the emergence of “hot” strains, which can overcome husbandry interventions. The increasing heat and stresses on fish populations due to global climate change are shifting the window of vulnerability to IHNV, calling for a change in prophylactic measures and vaccine design strategies.

For decades, laboratory work has been conducted on the viral lifecycle and host–pathogen interactions, but the findings have not been translated into immunotherapies that are effective in the field [15,16]. This is due to technical and regulatory barriers. Management routines like ozone treatment of water and UV sterilization offer protection only within limited areas and are inadequate for addressing the inherent risk of viruses in open-net-pen or large-scale raceway systems. Vaccine development has thus become a cornerstone of the long-term strategy for IHNV control [17]. Initial efforts using inactivated whole-virus preparations, as well as live-attenuated strains, provided the first proof of concept for teleost immunization, but these approaches frequently encountered issues with inconsistent efficacy and safety concerns regarding residual virulence [18,19,20]. Over the course of the last two decades, knowledge has significantly improved regarding the effect of viral pathogens on salmonid fish. Molecular vaccinology, particularly the development of DNA vaccines coding for the viral G protein, which contains many antigenic regions and epitopes (Figure S1 and Table S1) and is an important pathogenic factor for IHNV in inducing neutralizing antibodies in fish, has given us the confidence that salmonids can protect themselves for life if injected with the appropriate antigen [21,22]. Nonetheless, a significant delivery gap persists. While intramuscular injection can effectively deliver an antigen, its administration is labor-intensive and impractical for the millions of small-sized fries that are most susceptible. As a result, the current focus of research is on the development of oral and immersion delivery systems [23,24], the discovery of new adjuvants to stimulate mucosal immunity, and the application of reverse genetics to develop safer and more effective vaccine candidates [2,25,26]. Details on the current research and development status and immune efficacy of IHNV vaccines are presented in Table S2. The objective of this review is to bring together the above-mentioned themes in a single article to provide an overview of our current understanding of salmonid health and the steps necessary to secure a sustainable future.

## 2. Viral Architecture and Immunogenic Targets

The classic bullet-shaped structure of Rhabdovirus, which includes the family of IHNV, contributes to the virion’s stability and infectivity, measuring 150–190 nm in length and 65–80 nm in diameter [13]. The ribonucleoprotein core architecture of the virus contains an 11 kb single-stranded, negative-sense RNA genome that is tightly encapsulated by the nucleoprotein (N). The N protein not only serves as a structural scaffold but also functions as a switch between transcription and replication [27]. Furthermore, it protects the viral RNA from host ribonucleases (RNases) and maintains the viral genome in a template-active conformation for the viral RNA-dependent RNA polymerase (RdRp) [27]. The RdRp complex comprises a large (L) protein and the phosphoprotein (P, formerly referred to as M1). The L protein is a multifunctional catalytic giant with roles in RNA synthesis, capping, and polyadenylation [28]. The P protein serves as a bridge between the L protein and N-RNA template [3]. The matrix (M) protein (previously referred to as M2) encases the RNP core, facilitating condensation into a tightly coiled “skeleton”, and coordinates the budding process at the host cell’s plasma membrane [29]. Additionally, the M protein is a significant virulence factor that inhibits host cellular transcription, thereby preventing the fish from mounting an early antiviral response [30]. The entire assembly is enveloped by a host-derived lipid bilayer, with the most immunologically important component, the glycoprotein (G), protruding as spikes in a trimeric form [29].

The only surface protein and primary driver of viral entry for IHNV is the glycoprotein (G), which serves as the target of nearly all current vaccination strategies [31]. Functionally, the G protein, responsible for the fusion of most RNA viruses, is an integral membrane protein that undergoes significant conformation in a pH-dependent manner. This pH-dependent alteration occurs during endosome membrane fusion with the viral envelope [32]. Consequently, the G protein is essential for the fusion of the viral envelope with the membrane of the host cell membrane. It is synthesized as a precursor and undergoes extensive post-translational modifications, including N-linked glycosylation [32,33], which are essential for proper folding, stability, and “masking” some of the epitopes from the B-cell receptors of the host. The epitope of the fish that neutralizes the G protein has a structure that is recognized by the adaptive immune system. The utilization of monoclonal antibodies in research has led to the identification of at least three different antigenic sites on the IHNV G protein, with Site II being the main site for protective humoral responses. Since the G protein is the sole target for neutralizing antibodies (nAbs), the sequence conservation of G proteins across different genogroups is currently under close scrutiny. Even slight alterations in the amino acids of the G protein’s hydrophilic domains can induce antigenic drift, potentially rendering the virus unrecognizable to vaccines originating from heterologous strains [18,34]. Therefore, understanding the 3D structure and glycosylation patterns of the G protein is essential for the design of “epitope-focused” vaccines that could provide broad-spectrum protection.

Beyond the regular structural proteins, the genes that encode the NV proteins located in between the G and L genes are unique. Even though the NV protein does not make up part of the mature virion, it has a unique function as the “biological architect” of the infection milieu. This makes it a key, albeit indirect, target in the design of an attenuated vaccine. The NV protein primarily serves as an IFN antagonist; it accumulates in the nucleus and mitochondria of infected fish cells to prevent the activation of the RIG-I-like receptor (RLR) signaling pathway [35]. The NV protein gently refuses the phosphorylation and nuclear translocation of IRF3 and IRF7, which are required to increase interferon activity; as a result, the interferon alarm of the host cell remains silent during the early stages of viral replication [35,36]. Genetic deletions of the NV gene produce highly attenuated phenotypes that cannot cause disease but still express the highly immunogenic G protein. Therefore, ΔNV strains represent ideal candidates for live-attenuated vaccine platforms [2,37,38]. Furthermore, the interaction between the structural M protein and the non-structural NV protein provides the virus with a dual defense against host immunity. The M protein’s global inhibition of host protein synthesis complements the NV protein’s targeted disruption of the interferon system [18,39]. With this hierarchy of protein function in mind, vaccinologists can expand their focus from merely delivering antigens to developing vaccines that can effectively neutralize the virus.

## 3. Current Vaccine Modalities and Protective Efficacy

IHNV vaccines exhibit traditional whole-pathogen characteristics, and they can be categorized into two classes: inactivated (killed) and live-attenuated vaccines [7,17,23,24,40,41]. Inactivated vaccines, typically produced through the chemical cross-linking of viral capsids using formalin or beta-propiolactone, were the first to demonstrate that salmonids can achieve a certain level of humoral protection against a rhabdovirus challenge. These vaccines are considered reasonably safe, as they lack replicative potential; however, their protective efficacy is generally suboptimal due to the denaturation of crucial conformational epitopes on the G protein during the inactivation process. Research has indicated that inactivated IHNV vaccines can induce plaque-neutralizing antibodies and a specific group of pro-inflammatory cytokines. In contrast, live-attenuated vaccines are developed through serial passage in non-host cell lines or via chemical mutagenesis, allowing them to closely mimic natural infection and elicit a broader spectrum of immune responses. Consequently, they activate more innate and adaptive immune pathways, including significant cell-mediated CTL responses. Nonetheless, safety concerns, particularly the reversion to virulence and the potential for vaccinated fish to asymptomatically shed the virus for extended periods, largely restrict the commercial use of live IHNV vaccines. Despite these concerns, live-attenuated strains have proven to be valuable tools for defining the molecular mediators of protection, providing evidence that early induction of the IFN system is the strongest predictor of survival during an acute IHNV outbreak.

The advent of nucleic acid-based vaccines in the late 1990s revolutionized the control of IHNV, leading to the development of DNA vaccines [42,43], such as Apex-IHN^®^, which is today’s best prophylactic tool by far for salmonid aquaculture [44,45]. These vaccines use a bacterial plasmid as a vector, and this carries the IHNV G-protein gene under the regulation of a strong eukaryotic promoter, such as the human cytomegalovirus immediate early promoter (CMV promoter). Intramuscular injection of the plasmid leads to its uptake by myocytes and resident antigen-presenting cells, which synthesize and present the viral G protein. This process ensures that the antigen is properly folded and N-linked-glycosylated, thereby circumventing the limitations associated with protein-based vaccines. DNA vaccines against IHNV are likely the most effective engineered vaccines available, with laboratory trials consistently demonstrating Relative Percent Survival (RPS) values exceeding 95%. Notably, protection in trout occurs as rapidly as four days post-vaccination, which is attributed to a sustained non-specific “early antiviral phase” mediated by the Mx protein and other Interferon-Stimulated Genes (ISGs) [22,45,46,47,48,49,50]. However, despite their effectiveness, the efficacy of DNA vaccines is closely linked to the injection route, creating a logistical “delivery wall” for mass vaccination of fry and small fingerlings, which show the highest mortality rates from IHNV [51,52,53,54].

Due to the limitations of injectable platforms, the focus of IHNV vaccinology has shifted to oral and immersion delivery methods utilizing various biotechnological carriers such as algae, yeast, and encapsulated nanoparticles. The goal of the next generation of delivery routes is to bridge the delivery gap by non-invasively stimulating mucosal immunity in the gut, skin, and gills, which are the primary entry points for IHNV. Revolutionary research has developed *Pichia pastoris* as bio-factories for producing and co-encapsulating the recombinant IHNV G protein to protect it against the degradative environment of the fish gut [31]. In the past, early oral subunit vaccines exhibited low immunogenicity. However, the incorporation of a molecular adjuvant, such as a cytosine–phosphate–guanine (CpG) oligodeoxynucleotide or flagellin, enhances the activation of the teleost’s mucosal-associated lymphoid tissue (MALT) [55,56,57]. Furthermore, reverse genetics has enabled the development of these “designed” attenuated strains by deleting specific virulence genes (e.g., NV), resulting in the development of a vaccine virus that is capable of replication and provides high-level protection without causing clinical disease or persistence in the environment. Evaluations of multivalent oral formulations are increasingly being conducted in the field, balancing the absolute efficacy of DNA vaccines with the necessity for scalability in modern, high-density aquaculture systems [58,59]. The success of these approaches will depend on their ability to induce systemic IgG-like (IgY) and secretory IgT responses, which constitute the mucosal first line of defense against rhabdovirus.

## 4. Advanced Construction Models for Next-Generation Vaccines

The design of IHNV vaccines has evolved to encompass not only the direct exposure of antigens but also complex, multicompartmental delivery systems [2,7,60]. A particularly promising construction model involves viral vectored platforms leveraging the natural infective machinery of non-pathogenic or heterologous viruses to deliver IHNV immunogens. Reverse genetics systems have enabled the use of Viral Hemorrhagic Septicemia Virus (VHSV) as a backbone for constructing chimeric non-rhabdoviruses that expresses the IHNV glycoprotein in place of or in addition to their own surface proteins [61,62,63,64]. This construction model allows the vaccine candidate to retain its ability to replicate at low temperatures and infect the mucosal surfaces of fish, all while lacking the virulence of IHNV. Moreover, the combination of an adenovirus and a baculovirus vector is garnering attention [65,66]. Both systems serve as highly effective “gene delivery vehicles”, which can be easily engineered to display the IHNV-G protein on their surface or to subsequently express it within the cell. The intricate design of these systems facilitates the effective delivery of “molecular adjuvants” such as teleost cytokines (IL-1beta, TNF-alpha, or IFN-gamma) encoded in the same vector [67]. Scientists are achieving levels of mucosal stimulation that surpass those reached with traditional subunit or inactivated vaccine models by constructing vaccines that provide both the antigen and the “danger signal” necessary to activate the teleost immune system.

In the field of IHNV vaccinology, the construction model known as “Molecular Farming” represents a disruptive innovation parallel to the use of viral vectors. This model utilizes plant and algal systems to express and package viral proteins. Specifically, the IHNV-G gene can either be integrated into the chloroplast genome of microalgae such as *Chlamydomonas reinhardtii* or expressed in the leaves of *Nicotiana benthamiana*. This method offers advantages to produce a cost-effective and efficient “bioreactor”, as well as a protective “bio-capsule”. The recombinant G protein is shielded from the damaging acidic environment and the enzymes present in the fish stomach by the rigid cellulose walls of the algal or plant cells [68,69]. Consequently, the resulting antigen reaches the hindgut, as this is the primary area for teleost mucosal immune induction. Additionally, this approach allows for the production of virus-like particles (VLPs) [70], which, despite lacking a viral genome, self-assemble to mimic IHNV morphology. By presenting the G protein in its natural, trimeric, high-density configuration on the VLP surface, these constructs elicit a significantly stronger B-cell response than the soluble, monomeric proteins produced in *E. coli*. The creation of these “nanoscaffolds” exemplifies the pinnacle of structural bioengineering [71,72], providing a blueprint for developing oral vaccines that can withstand room temperature and be produced on a large scale for the global salmonid industry.

The latest and most advanced construction model utilizes mRNA and nanoparticle encapsulation, which has rapidly evolved since the successful implementation of human mRNA platforms [73]. IHNV necessitates the in vitro transcription of a stabilized mRNA coding for the G protein, followed by its encapsulation in lipid nanoparticles (LNPs) or polymeric nanoparticles such as chitosan or PLGA (poly lactic-co-glycolic acid). The design of these LNPs involves a meticulous thermodynamic process that enhances stability, ensuring that the mRNA is protected from extracellular RNases while facilitating endosomal escape after particle uptake by fish cells. A significant subdivision of this model is the development of “replicon” vaccines based on the genome of the alphavirus [74,75,76]. The IHNV-G gene can be engineered in place of the alphavirus structural genes to generate a self-amplifying RNA (saRNA) [77,78]. When this saRNA enters a fish cell, it instructs the cell to manufacture thousands of copies of the IHNV-G mRNA. This production of numerous copies of the antigen is achieved from a very low dose. This “amplification loop” not only prolongs the exposure of the antigen but also acts as an internally generated adjuvant; the double-stranded RNA intermediates produced during replication serve as potent activators of fish Toll-like receptor 3 (TLR3) and RIG-I [79,80].

## 5. Technical Bottlenecks in IHNV Vaccinology

In the field of IHNV vaccination, the most significant technical challenge currently faced is often referred to as the “Immune Ontogeny Paradox.” This paradox highlights the unexpected disconnect between peak host susceptibility, which occurs in fry, and the developmental maturation of the teleost adaptive immune system. IHNV causes the most severe losses in fry and small fingerlings (usually <1.0 g). However, the adaptive repertoire of salmonids, characterized by the diversification of B-cell receptors, and the full functional capacity of MALT are not fully operational until several weeks later. This results in the creation of a “window of vulnerability” due to traditional vaccine-induced responses not leading to lasting immunity. Additionally, it has been documented that maternal transfer of IgM through the yolk may provide transient protection, but it can also interfere with active vaccination, leading to “maternal antibody interference” [81]. Therefore, developing a vaccine that can circumvent this immaturity to stimulate the neonatal innate system by inducing persistent activation of the TLR22 or retinoic acid-inducible gene I (RIG-I)-like pathways will require a level of precision in adjuvant formulation that may be challenging to achieve commercially [82]. Until the developmental hurdle is overcome, the industry remains trapped in a cycle of managing diseases reactively rather than proactively.

What this means in practice is that, beyond host physiology, a significant “delivery–efficacy gap” exists for many vaccine types. This gap is particularly evident for injectable DNA vaccines when transitioning to mucosal platforms [83]. When the same antigenic constructs are employed for immersion and oral application, their efficiency declines sharply due to “mucosal degradation” and “antigenic dilution” [84]. In the aquatic environment, vaccine particles must be resilient to changing pH, be resistant to enzymatic degradation in the water column, and, above all, be capable of penetrating the mucus barrier of fish before reaching the epithelial cells for uptake. The dose required for effective oral immunization is often greater than that needed for injection, resulting in economically prohibitive scaling [85]. Furthermore, the teleost gut exhibits oral tolerance [86,87]. This is a physiological mechanism that prevents an inflammatory immune response to innocuous non-self-antigens following oral administration. Unfortunately, this can lead to a systemic tune-out of the vaccine, making fish more, rather than less, susceptible to subsequent IHNV challenges.

The evolving regulatory landscape constitutes a “moving target” bottleneck for the licensure of next-generation IHNV vaccines. While DNA vaccines have proven to be safe, their classification as Genetically Modified Organisms (GMOs) in important markets such as the European Union continues to restrict their use and fragment trade. The rapidly changing IHNV genogroups, which undergo prominent “antigenic drift”, further complicate this regulatory challenge. A vaccine based on a U genogroup isolate may exhibit significantly reduced neutralization titers against the J or M variants that emerged in 2026 [88,89]. This situation necessitates the development of “multivalent” or “consensus” G-protein sequences, which complicates the molecular design and regulatory filing of the vaccine [65]. Furthermore, there is no standard correlate of protection, so researchers must rely on costly and ethically complex “live challenge” trials to prove efficacy. No biomarker, such as a serum antibody titer, is universally accepted by all as guaranteeing survival in the field. The pathway for innovative IHNV vaccines will remain constrained by administrative rather than biological factors until the global community establishes high-throughput, in vitro assays for vaccine validation and harmonizes the regulatory status of nucleic acid technologies.

## 6. Deficiencies and Limitations of Existing Research

A significant gap in current IHNV research is the emphasis on systemic RPS, which is not complemented by an understanding of mucosal transmission and shedding. Recent trials involving DNA vaccines and inactivated vaccines frequently report a high level of protection against lethal challenges [7,90]. However, in 2025, it was demonstrated that these protected survivors still shed substantial amounts of infectious virions into water. This phenomenon of “leaky vaccines” indicates that current models are insufficiently effective in stimulating immune responses that adequately protect the gills and skin from sterile entry and exit [7,91]. Furthermore, there is a lack of quantitative estimation studies to determine the epidemiological consequence of this residual shedding in high-density cage environments, where even vaccinated stocks may serve as virus reservoirs, potentially infecting wild salmonid stocks or unvaccinated populations. In addition, since no standard protocol exists for measuring viral titers in water (environmental DNA/RNA) after challenge, we have substantial knowledge regarding how vaccines protect individual fish but little understanding of their impact on “herd immunity” and viral loads in the whole watershed [7,91,92].

Another major limitation is the “temporal myopia” of immunological assays, where long-term cellular memory remains a poorly characterized “black box”. Most evaluations of IHNV vaccines are completed within 90 to 180 days post-vaccination, but commercial salmonid production cycles extend beyond two years [90,93]. Our understanding of the teleost head kidney’s memory niche and factors regulating the longevity of plasma cells and memory T cells is limited [94,95]. Although the persistence of DNA vaccines is generally acknowledged, the precise molecular signals that transform an early, non-specific interferon response into a stable, long-lasting adaptive response remain debated. Furthermore, the lack of research on the “exhaustion” of the fish immune system due to fluctuating temperatures or secondary infections complicates predictions regarding when a “booster” may be necessary [96]. The increasing recognition that neutralizing antibody titers cannot be relied on as a sole correlate of protection, as decoupling is frequently reported between neutralizing antibody levels and survival, suggests that cell-mediated immunity (CMI) plays a significantly larger role than indicated in standard ELISA or plaque reduction assays [97,98,99].

In summary, there exists a systemic failure in research to globally address “antigenic breadth” and cross-genogroup protection in a standardized manner. Although there is a considerable amount of published literature on this topic, it appears to be siloed geographically. For instance, East Asia focuses on genogroup J, North America focuses on U and M, and Europe focuses on the E group. It is quite apparent that there is no obvious “pan-genogroup” study evaluating whether the vaccine candidate from a lineage can generate sufficient “sterilizing immunity” against an emergent heterologous variant. This is aggravated by the absence of a universal “reference strain” of IHNV [53,100,101], which renders it nearly impossible to compare the outcomes of a subunit vaccine trial in Chile with those of a viral vector trial in Norway. Additionally, there is limited understanding of the host genetic–vaccine interaction, particularly regarding whether the differences in MHC genes between rainbow trout and Atlantic salmon, when reacting to the same IHNV G-protein epitope, contribute to genetically driven variations in vaccine efficacy [102,103].

## 7. Future Perspectives: The Path to “Smart” Vaccines

A shift from a “reactive” vaccine design towards a more “predictive” vaccine design for IHNV prophylaxis is achievable through the utilization of Artificial Intelligence (AI) and machine learning (ML). Currently, researchers employ deep learning algorithms to map the fitness landscape of the IHNV G protein, predicting which amino acid substitutions will be favored in neutralizing epitopes under vaccine-driven selection. By adopting such “in silico” models, scientists can develop consensus or mosaic antigens, which are synthetic proteins capable of representing the entire range of U, M, L, E, and J genogroups [5,104]. The smart antigens engineered by researchers are designed to optimally activate broadly neutralizing antibodies (bnAbs) that target highly conserved structural motifs in the relevant viruses, such as the pH-sensitive fusion loop. The evolution of the virus is constrained in mutating such motifs due to the risk of losing infectivity. Furthermore, the integration of immunoinformatics enables the identification of T-cell epitopes that are compatible with the broad MHC Class I and II alleles of different salmonid populations. This personalized approach to aquaculture vaccinology ensures that certain formulations can effectively protect genetically diverse species and genetic lines, thereby bridging the gap caused by antigenic drift and host genetic diversity.

Next-generation IHNV vaccines may leverage self-amplifying RNA (saRNA) [105] and programmable nanoparticles to address challenges related to delivery and durability as technology advances. Unlike mRNA, which requires a ribosome for replication, saRNA contains the genetic code for a viral replicase (typically derived from alphaviruses) and can replicate independently once inside the cell. This “amplification loop” is exceptionally beneficial for the mass immersion vaccination of fry, as it generates a substantial and sustained antigenic signal from a significantly reduced initial dose. When incorporated into smart stimuli-responsive nanocarriers, these RNA payloads can be programmed to release upon physiological triggers, such as in the alkaline environment of the fish hindgut or specific cellular uptake processes in the fish gills. Furthermore, these nanoparticles can be loaded with ligands targeting dendritic-like cells or antigen-presenting cells (APCs) on the fish’s mucosal surfaces, ensuring that the vaccine is not only absorbed but also effectively transported to lymphoid tissues. This precision engineering facilitates the efficient initiation of viral infection, allowing for optimal payload delivery with maximum safety using precisely designed viruses. It presents a scalable solution to the “delivery–efficacy gap” that has hindered the industry for years.

The shift towards systemic mucosal priming [106,107] with the assistance of molecular adjuvants and trained immunity represents a frontier in the control of IHNV. Future “smart” vaccines are likely to incorporate TLR agonists and small molecules that induce interferon production [108,109], which will be tailored to the kinetics of the salmonid immune response. By co-delivering these molecular triggers alongside the IHNV G protein, researchers can leverage the fry “Immune Ontogeny Paradox” to elicit an innate antiviral state while the adaptive immune system is still developing. Research focusing on “trained innate immunity” [110], where exposure to non-specific ligands (such as β-glucans or specific viral particles) “reprograms” the epigenetic state of myeloid cells to a more alert state capable of combating a range of pathogens, not just IHNV, is gaining traction. As we enter an era of “precision aquaculture,” the application of these vaccines will be coordinated with real-time environmental sensing and environmental DNA (eDNA) monitoring. This approach would facilitate “precision-strike” vaccination, allowing for a formulation tailored to the circulating strain, and the thermal profile of the farm could be applied at the time of the highest outbreak risk. The advent of the “smart” vaccine era promises to transform IHNV from a potential catastrophe into a manageable component of sustainable salmonid farming, bridging the gap between advanced molecular biology and field environmental data.

## 8. Discussion

Since its initial characterization in the mid-20th century, infectious hematopoietic necrosis virus (IHNV) has emerged as a widely distributed pathogen in aquaculture. This situation underscores the challenges posed by modern intensive farming practices. As discussed in this review, the battle against IHNV is now being waged on multiple fronts, necessitating a collaborative approach involving molecular virology, vaccinology, and ecology. We have evolved from the use of “brute force” vaccination—characterized by inactivated whole-virus preparations that demonstrated inconsistent efficacy—to more advanced DNA and mRNA platforms that can induce selective and long-lasting immunological memory. The pG-DNA vaccine represents a significant milestone in aquatic medicine, demonstrating that at least one fish species can respond to high-quality DNA in a sophisticated eukaryotic-like manner. However, the “paradox of the DNA vaccine”—where its high efficacy is countered by delivery challenges—continues to represent a research bottleneck in the field. The future of the industry will hinge on the successful translation of injected systemic protection into scalable, non-invasive formats such as oral and immersion methods.

The development of next-generation construction models indicates that bio-encapsulation, along with viral vector chimeras, is effectively bridging the delivery gap. Microbial virus-like particle (VLP) systems derived from *Pichia pastoris*, microalgae, or plant-based systems function as living capsules that adeptly navigate the hostile physiological environment of the teleost digestive tract [111,112]. These models not only provide an antigen but also deliver a substantial “biological package” that safeguards the structural integrity of the G protein and offers the necessary co-stimulatory signals to MALT. The research landscape of 2026 emphasizes the importance of trained immunity and molecular adjuvants [17]. Furthermore, the fish’s innate system (i.e., the RIG-I and TLR22 pathways) serves as more than just a first line of defense [113,114,115]. Rather, it is a critical gatekeeper for adaptive memory. Altering the vaccine to the specific ontogeny of the salmonid immune system should address the vulnerability of fry and fingerlings, ensuring protection is conferred prior to their first exposure to waterborne virions.

However, it is crucial for the scientific community to remain vigilant regarding the aspects that we do not fully understand. The phenomenon of “leaky vaccines” and the ongoing shedding of IHNV by vaccinated survivors pose a significant epidemiological risk that could undermine “herd immunity” in open-water cage systems. Leaky IHNV vaccines reduce mortality but allow for subclinical infections and persistent viral shedding, which facilitates viral circulation within vaccinated populations. This phenomenon sustains environmental reservoirs, promotes genetic drift, and increases the risk of spillover to wild salmonids, ultimately undermining long-term control efforts. Furthermore, high-dose exposure exacerbates viral shedding, thereby worsening transmission dynamics.

Future research should prioritize the development of sterilizing immunity combined with integrated management (fallowing, stress reduction, and genetically resistant stock to lower transmission pressure) and biosecurity (surveillance and disinfection), focusing not only on preventing mortality but also on halting viral replication on mucosal surfaces. This will require a deeper understanding of secretory IgT [116,117,118] and the contribution of resident memory T cells in the gills and skin [119]. Furthermore, it is essential to develop consensus and mosaic vaccine designs to address the global fragmentation of IHNV genogroups. As IHNV evolves in response to climate change and intensified production practices, our vaccines must also undergo “smarter” adaptations, utilizing AI-driven epitope prediction to account for the virus’s natural antigenic drift.

## 9. Conclusions

As discussed, the path to a “world without IHNV” is not linear; instead, it must integrate both technology and policy. We must advocate for the global harmonization of standards regulating nucleic acid-based vaccines and GMO-labeled technologies to enable the most effective tools to be deployed in areas where they are needed most. The objective is precision aquaculture, which utilizes eDNA in real time to deliver tailored multivalent vaccines. Through molecular precision, oral delivery logistics, and machine learning predictability, the future of salmonid aquaculture can be secured using saRNA platforms. This review serves as both a record of our advancements and a strategic plan; IHNV remains an ever-present adversary. Nevertheless, modern biotechnology is providing us with the clearest path yet toward sustainable long-term viral control. The next decade will be defined by our ability to translate these laboratory breakthroughs into the raceways and sea cages that will feed the world.

## Data Availability

The original contributions presented in this study are included in the article and Supplementary Materials. Further inquiries can be directed to the corresponding authors.

## Supplementary Materials

The following supporting information can be downloaded at: https://www.mdpi.com/article/10.3390/vaccines14040314/s1, Figure S1: The pattern diagram of IHNV-G protein antigenic regions and epitopes; Table S1: Antigenic epitope regions of the IHNV G protein; Table S2: The profile of IHNV vaccine type and immune efficacy. References [120,121,122,123,124,125,126] are cited in the Supplementary Materials.
