# Supplementary material for: Advancements in Vaccinology Against Infectious Hematopoietic Necrosis Virus (IHNV): From Traditional Methods to Next-Generation Strategies
Source: Vaccines (Basel). 2026 Mar 31;14(4):314. doi: 10.3390/vaccines14040314 (PMC13119925; doi:10.3390/vaccines14040314)
Supplement: Supplementary file 1 [file vaccines-14-00314-s001.zip › vaccines-4194473-supplementary.pdf]

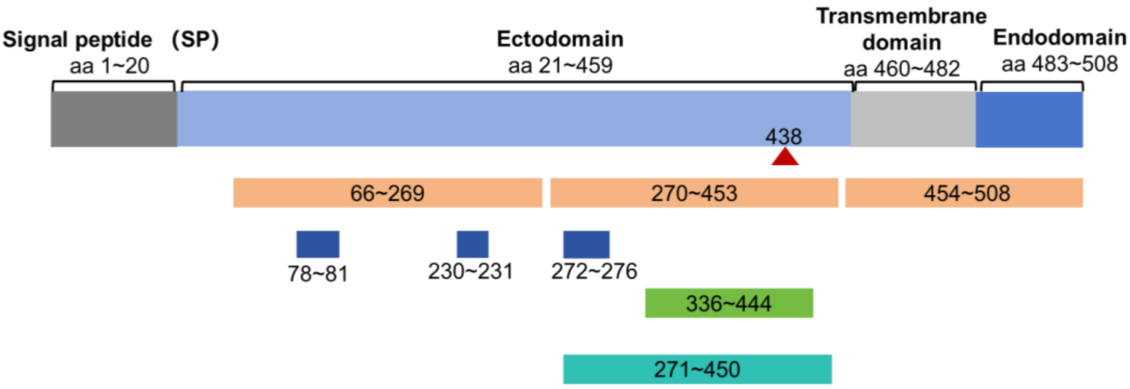

Figure S1. The pattern diagram of IHNV-G protein antigenic regions and epitopes. It illustrates the starting and ending boundaries of the antigenic regions, as denoted by the numbers in the figure. This representation aids in the understanding of the spatial organization of these critical immunogenic components.

Table S1. Antigenic epitope regions of the IHNV G protein.

Antigenic regions/sites[a]	Function	Reference
78-81,230-231,272-276	Neutralizing antibody	[120]
66-269,270-453,454-508	Neutralizing antibody	[121]
336-444	Neutralizing antibody	[122]
271-450	Neutralizing antibody	[123]
438	Achieving immune evasion	[124-126]

[a] IHNV WRAC strain (GenBank:KP216199)

Table S2. The profile of IHNV vaccine type and immune efficacy.

Type	Status	Primary Route(s)	Typical Efficacy (RPS)
DNA Vaccine	Commercial	Intramuscular (IM)	90% – 100%
Live Attenuated	Experimental	Immersion/Intranasal	70% – 98%
Inactivated (Killed)	Experimental	Intraperitoneal (IP)	30% – 60%
Recombinant Subunit	Experimental	IP/Oral	40% – 80%
